# Capturing context-specific regulation in molecular interaction networks

**DOI:** 10.1186/s12859-018-2513-7

**Published:** 2018-12-22

**Authors:** Stephen T. A. Rush, Dirk Repsilber

**Affiliations:** School of Medical Sciences, Örebro University, Södra Grev Rosengatan, Örebro, Sweden

**Keywords:** Activated subnetwork, Coherent differential expression, Differential regulation, Error control, Functional module, Molecular network

## Abstract

**Background:**

Molecular profiles change in response to perturbations. These changes are coordinated into functional modules *via* regulatory interactions. The genes and their products within a functional module are expected to be differentially expressed in a manner coherent with their regulatory network. This perspective presents a promising approach to increase precision in detecting differential signals as well as for describing differential regulatory signals within the framework of a priori knowledge about the underlying network, and so from a mechanistic point of view.

**Results:**

We present Coherent Network Expression (CoNE), an effective procedure for identifying differentially activated functional modules in molecular interaction networks. Differential gene expression is chosen as example, and differential signals coherent with the regulatory nature of the network are identified. We apply our procedure to systematically simulated data, comparing its performance to alternative methods. We then take the example case of a transcription regulatory network in the context of particle-induced pulmonary inflammation, recapitulating and proposing additional candidates to previously obtained results. CoNE is conveniently implemented in an R-package along with simulation utilities.

**Conclusion:**

Combining coherent interactions with error control on differential gene expression results in uniformly greater specificity in inference than error control alone, ensuring that captured functional modules constitute real findings.

## Background

**Fig. 1:**
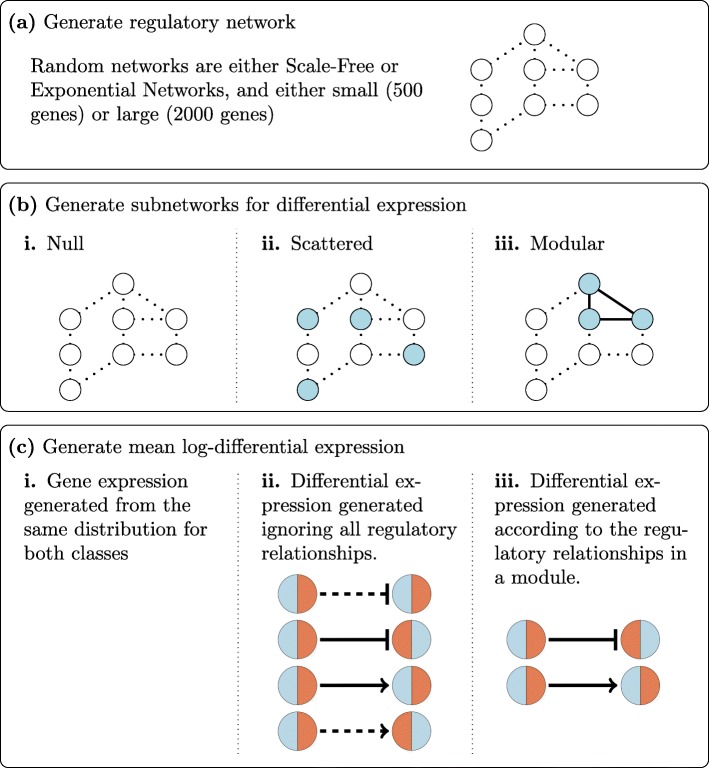
Summary of Gene Expression Simulation. **a** Regulatory networks are randomly generated. Circles: molecular entities, e.g. genes; Lines: principle molecular regulatory interactions (links). **b** Genes are randomly selected to exhibit differential gene expression according to whether differential expression is i. null, ii. scattered, or iii. modular. **c** Mean log-expression is generated according to whether differential expression is i. null, ii. scattered, or iii. modular

Molecular profiles reveal how for example gene expression changes over time and in response to perturbation events, for example changes in environmental gradients. These changes are coordinated *via* regulatory interactions. Regulatory interactions form a network of potentially active links between genes (Fig. 1a). Differentially expressed genes are expected to have neighbours that are differentially expressed (Fig. 1b iii), rather than scattered about the network at random (Fig. 1b ii). We further expect these neighbourhoods to be coherent with the regulatory relationships. In this article we identify differentially expressed subnetworks coherent with the regulatory structure, achieved by integrating differential gene expression with the associated network. Gene expression is routinely measured at the level of expressed RNA transcripts for each gene. Differentially expressed (DE) genes are those genes exhibiting a change in mean gene expression between conditions. However, genes do not act in isolation. Rather, they act in biological networks consisting of interacting coordinated modules and more loosely coupled super-modules [1]. Ravasz et al. [2] first demonstrated this empirically in organisms spanning the three domains of life, finding that their metabolic networks are organized into highly connected modules, which are then more loosely coupled in a hierarchical fashion. The molecules within a functional module are expected to be differentially regulated in a coherent manner, i.e. respecting the regulatory network structure, in response to changes in their environment. From a systems level perspective, molecular entities, e.g. genes, always act together in pathways and modules. The behaviour of these interactions aid in the study of the functions of genes and their products. For example, coordinated changes may be captured by gene co-expression patterns, which measure correlations. Use of direct correlations results in many false positives, and various methods exist to correct this [3, 4]. More recent methods profit from prior topological knowledge to constrain inference in network regulation. Specifically, there is an emphasis on context-specific network regulation [5–8]. Numerous network-based regularization methods profiting from previous studies have emerged to perform variable selection and to obtain biologically meaningful predictors [9–11]. Ma et al. [12] perform gene set enrichment analyses using either complete or incomplete topological information. These methods assume that a functional pathway is differentially active if most genes in this network structure are differentially expressed.

In this article, we emphasize regulatory coherence. Regulatory coherence refers to gene expression patterns that respect the regulatory nature of the network. A network is described by a graph *G*=(*V*,*E*) for a set *V* of vertices and a set *E* of edges between vertices. For a gene regulatory network (GRN), the vertices represent genes while the edges indicate interactions between genes, such as activation and inhibition. We will refer to edges and vertices as links and genes, respectively. Inducing and inhibiting links are called regulatory links. Each gene regulates or is regulated by genes in its network topological neighbourhood. We define **coherent differential expression (CDE)** as the tandem changes in gene expression for a pair of genes in a link that is consistent with the regulatory nature of the link. We distinguish between inhibitory and non-inhibitory links. Non-inhibitory links consist of inducing links, relationships without explicit direction such as binding, or positive correlations where the regulatory relationship is unknown. Tandem changes in gene expression for an inhibition link are coherent if, as the expression of gene *A* increases, the expression of gene *B* decreases. In contrast, CDE for non-inhibitory links occurs when the expression of both genes increases or decreases. Outside these two cases, differential expression is said to be incoherent. There are many reasons an interaction could be incoherent. First and foremost, coherent differential expression captures signals that dominate the network; some interactions are dynamically promoted while neighbouring interactions are dynamically demoted. Additionally, incoherent DE could point to issues within the underlying network model. For example, if DE has occurred via some phenomenon not represented in the network (e.g. we look only at a GRN but some non-GRN event occurs), this informs us that our model is too simple. Indeed, the incoherence of an expected interaction can point to non-canonical pathways.

With regulatory coherence, it becomes clear that a GRN represents a collection of potential interactions, which are realized in specific contexts and which can be related to observed changes in expression. These realized interactions form the **coherent subnetwork**. We present Coherent Network Expression (CoNE), a procedure for identifying coherent expression together with error control. This combination is to increase precision in identifying functional modules in molecular interaction networks. We systematically evaluate CoNE through comparison with other methods for identifying differential expression in networks, using simulations where the ground truth is known. Once validated, we apply CoNE to the problem of identifying differentially expressed subnetworks in an in vitro pulmonary inflammation gene expression study.

## Methods

### Coherent differential expression

In this section, we make our concept of coherence precise and describe our procedure, Coherent Network Expression (CoNE), for identifying coherent subnetworks. Let *G* be a network with gene set *V*(*G*) and link set *E*(*G*), and let *S*⊆*G* be the coherent subnetwork corresponding to differentially expressed genes. The genes in the gene set *V*(*S*) are differentially expressed while all others, *V*(*G*)∖*V*(*S*), are not. The link set *E*(*S*) consists only of those links respecting regulatory coherence. Note that it is not necessary that all links between genes in *V*(*S*) be included.

We assign link weights *w* according to the relationship encoded in a link. For a link (*j*,*k*)∈*E*(*G*) between genes *j* and *k*, the link weight is defined as

1 $$\begin{array}{*{20}l} w(j,k) = \left\{\begin{array}{ll} \; -1 & \text{if the relationship is inhibiting} \\ \quad 1 & \text{otherwise.} \end{array}\right. \end{array} $$

#### Link coherence

We consider first the simple case where we have two experimental conditions, and we are interested in the differential behaviour between the two. For a gene *j*, let *δ*_*j*_ be the differential expression between the two conditions. Consider the link (*j*,*k*)∈*E*(*G*) between genes *j* and *k*, with relationship *w*(*j*,*k*). Then we say that link (*j*,*k*)∈*E*(*G*) is **coherent** if

2 $$\begin{array}{*{20}l} \text{sign}(\delta_{j}) \cdot \text{sign}(\delta_{k}) \cdot w(j,k) = 1. \end{array} $$

That is, when the relationship is normally inducing, then the differences *δ*_*j*_ and *δ*_*k*_ must agree on sign. Similarly, when the relationship is normally inhibiting, then the differences *δ*_*j*_ and *δ*_*k*_ must bear opposite signs. Otherwise, we say that the link is **incoherent**.

Consider now the general linear model

3 $$\begin{array}{*{20}l} \mathbb E(Y_{j}) &= \mu_{j} + \delta_{j} W + \beta_{j} Z,  \end{array} $$

where *μ*_*j*_ is the grand mean expression for gene *j*, *δ*_*j*_ is a vector of factor parameters for treatment vector *W*, and *β*_*j*_ is a vector of parameters for additional covariates *Z*. We can extend the definition of coherence to general linear models, where we are interested in specific contrasts *γ* of the factors in *W*, while controlling for other variables *Z*. For a gene *j*, let *δ*_*j*_(*γ*) be the parameter for the contrast *γ*. Then we say that the link (*j*,*k*)∈*E*(*G*) is **coherent with respect to contrast*γ*** if

4 $$\begin{array}{*{20}l} \text{sign}(\delta_{j}(\gamma)) \cdot \text{sign}(\delta_{k}(\gamma)) \cdot w(j,k) = 1. \end{array} $$

Otherwise, the link is **incoherent with respect to contrast*γ***. This definition easily accomodates the output from, for example, R packages lsmeans [13] for linear and mixed effects models or limma [14] for empirical Bayes estimation.

#### Coherent network expression (CoNE)

**Fig. 2:**
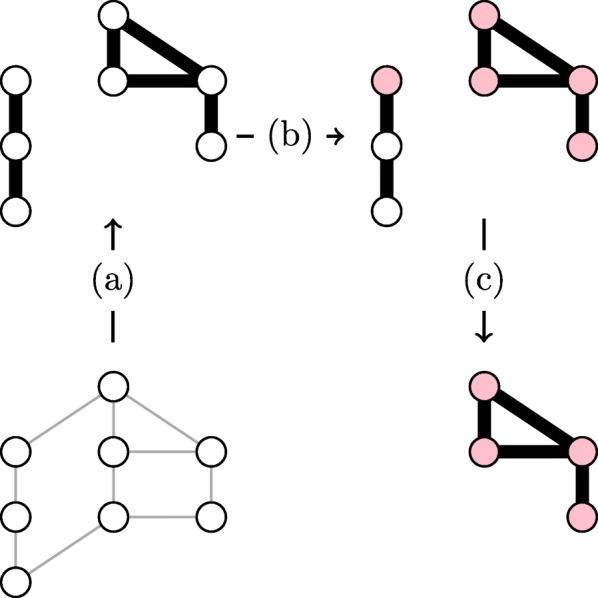
Coherent Network Expression Procedure. Beginning with a base regulatory network, **a** coherent links are identified and incoherent links are removed as well as isolated genes, **b** significant differentially expressed genes are identified, **c** all genes with non-significant differential expression and newly isolated genes are removed

We combine error control with link coherence in CoNE as follows (Fig. 2): **1.** We obtain estimates for models (3) across all genes. **2.** For a contrast *γ*, we obtain estimates $\hat \delta _{j}(\gamma)$ for all genes *j*∈*V*(*G*) in the network. **3.** We classify all links as coherent or incoherent and remove all incoherent links (Fig. 2a. Any genes that are isolated, i.e. with vertex degree 0, are also removed. In this way we enrich those genes in the coherent subnetwork. **4.** We assess significance of estimates $\hat \delta _{j}(\gamma)$ with false discovery rate (FDR) control (Fig. 2b, removing all genes with estimated FDR above the control threshold (Fig. 2c. We again remove all isolated genes.

In this paper, we use LIMMA [14] to fit all linear models and we adopt the Benjamini-Hochberg [15] false discovery rate procedure for error control in all our analyses.

**Fig. 3:**
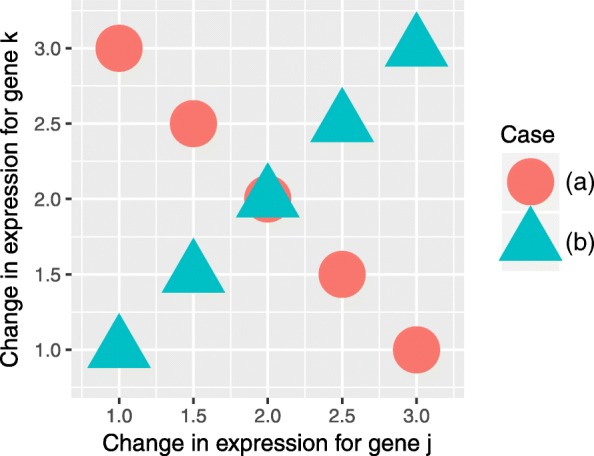
Example Differential Expression for an Inducing Link. The mean differential expression for genes *j* and *k* is 2 in both cases, and hence the same differential link score. However, in Case **a** the differential link score is constant (4) while in Case **b** the differential link score ranges from 2 to 6. Thus their differential link scores have different variance (0 versus 1.58), and hence Case **a** has greater statistical significance, whereas Case **b** exhibits the expected positive correlation for an inducing link

The initial inspiration for CoNE was the recent FocusHeuristics [7], which seeks to focus on the coherent subnetwork using three measures: mean absolute differential expression, differential link score, and interaction link score. The differential link score is a measure of the magnitude of the coherence between genes in a link, defined as the difference $\left (X_{j}^{1}+w(j,k)\cdot X_{k}^{1}\right) - \left (X_{j}^{0}+w(j,k)\cdot X_{k}^{0}\right)$ between cases 0 and 1. We have previously formalized hypothesis testing and error control of the differential link score (unpublished [16]). However, this approach suffers from two problems. The first is that incoherent expression can be identified as significantly coherent if the magnitudes of the differential expression between two genes in a link are sufficiently different. This is remedied by additionally ensuring coherence of the link, as in this article. The second problem is more fundamental, in that it penalizes those changes in gene expression that are highly correlated, as the expression for an inducing link illustrated in Fig. 3 demonstrates. In both cases depicted, average change in gene expression for both genes is 2, and hence their differential link score is 4. However, for Case (a), the differential link score is constant and so the variance of the score is 0, while for Case (b) the variance is 1.58. Thus the score in Case (a) is (infinitely) more significantly greater than zero than in Case (b). We see no reason we should favour Case (a) over Case (b). CoNE does not suffer from these problems.

#### Boundary of a subnetwork

Together with the notion of regulatory modules/subnetworks, it will be important to describe the boundary *∂S* of a subnetwork *S* in a network *G*. We define this here. Let *I* be the collection of links in *S* that are incoherent,

5 $$\begin{array}{*{20}l} I = \{(j,k)\in E(S): \text{sign}(\delta_{j}) \cdot \text{sign}(\delta_{k}) \cdot w(j,k) = -1\}, \end{array} $$

and let *B* be the collection of links in *G* with one gene in *V*(*S*) and the other in *V*(*G*)∖*V*(*S*),

6 $$\begin{array}{*{20}l} B = \{(j,k)\in E(G): &\quad(j\in V(S)\text{ and }k\in V(G)\backslash V(S))  \\ &\text{ or} (k\in V(S)\text{ and }j\in V(G)\backslash V(S))\}. \end{array} $$

Then the boundary *∂S* of the subnetwork *S* in *G* is defined as *∂S*=*I*∪*B*.

### Simulation and analysis of differential expression in networks

We develop simulations to evaluate the ability of our method to identify coherent interactions and DE modules. To model the dependence structure among genes, gene expression data is simulated as log-normally distributed according to a Gaussian Graphical Model. For each replicate, a random network *G*, covariance matrix *Σ* consistent with *G*, and mean log-expression vectors *μ*^0^ and *μ*^1^ are generated. Log-expression is sampled from models *N*(*μ*^*c*^,*Σ*), *c*=0,1. For each replicate, we consider sample sizes *n*=8,16,32, and 64, with 50% of samples in each class *c*. There are 100 replicates for each network type. The simulations are summarized in Fig. 1. We provide details in the following.

#### Gaussian graphical models

In our approach to simulate a regulatory network *G*, each node *j*∈*V*(*G*) interacts with a subset of the network, its neighbourhood *N*_*j*_={*k*:(*j*,*k*)∈*E*(*G*)}. Let *X*_*j*_ be the expression for a gene *j*. The expression *X*_*j*_ is anticipated by its neighbourhood: given the expression in neighbourhood *N*_*j*_, no further information is gained for the prediction of *X*_*j*_ by learning the expression of gene *l*∉*N*_*j*_, *l*≠*j*. In other words, nodes *j* and *l* are conditionally independent given *N*_*j*_. The joint distribution of gene expression may be factorized along the maximal cliques of the graph, and hence motivates the application of graphical models. We simulate differential gene expression using Gaussian graphical models (GGMs), which may be specified by their mean vectors and inverse covariance matrices [17]. Let *Σ*^−1^ be an *n*×*n* real positive definite matrix such that (*Σ*^−1^)_*jk*_=0 whenever (*j*,*k*)∉*E*(*G*) for *j*≠*k*. This implies that the partial correlation between genes *j* and *k* is zero for all non-linked genes in the network. Further let *μ* be fixed in $\mathbb R^{n}$. Then the distribution $\mathcal N(\mu, \Sigma)$ describes a GGM with corresponding network *G*.

#### Random graphs

In the simulations we use the following random networks: Exponential Erdős-Rényi and Scale-free Barabási-Albert [18]. (i) For the exponential graphs, we supply the following parameters for the number of genes and links (*v*,*e*): (500,2000), and (2000,8000). (ii) For the scale-free graphs, we set the number of genes, power of preferential attachment, and number of links to add at each time-step (*v*,*p*,*m*) as (500,1,2) and (2000,1,2). See R-package igraph for details [19].

The (first) Erdős-Rényi model considers an initial set of *v* genes, with *e* links chosen uniformly from the set of all *v*(*v*−1)/2 unique links between genes. Its topology is said to be exponential due to the distribution of vertex degree, which follows a Poisson distribution. On the other hand, the Barabási-Albert model belongs to the class of scale-free graphs, so called because there is no ‘typical’ node degree, with the degree distribution following an approximate power-law. Beginning with the biologically compelling assumption that as a network grows, new nodes attach preferentially to nodes with higher degree, [18] demonstrated that random graphs produced in this way are scale-free. Even though most biological networks appear to be scale-free, exponential graphs still arise naturally. Barabasi and Oltvai [1] mention for instance that *Saccharomyces cerevisae* and *Escherichia coli* exhibit mixed exponential and scale-free features, noting that the incoming degree distribution for transcription regulatory networks is approximately exponential while the degree distribution of transcription factor interactions is scale-free.

#### Differential expression and localization patterns

We investigate both null and true differential expression where genes are differentially expressed at random or in modules. For the small graphs (*v*=500), we simulate data where (i) there is no differential expression (null), (ii) differential expression is distributed randomly over the genes (low: 1% DE; high: 10% DE), (iii) differential expression is restricted to a connected subgraph (low: 1% DE; high: 10% DE), (iv) differential expression is restricted to three connected subgraphs (low: 3% DE; high: 30% DE) with average size 5 (low) and 50 (high). For the large graphs (*v*=2000), we simulate data where (i) there is no differential expression (null), (ii) differential expression is distributed randomly over the genes (low: 1% DE; high: 10% DE), (iii) differential expression is restricted to a connected subgraph (low: 1% DE; high: 10% DE), (iv) differential expression is restricted to three connected subgraphs (low: 1% DE; high: 10% DE) with average size 20 (low) and 200 (high). Expression patterns are depicted in Fig. 1b.

The generation of differential expression depends on the expression pattern. We generate mean log-expression vectors *μ*^0^ and *μ*^1^ for classes 0 and 1. The entries of *μ*^0^ are sampled from a uniform distribution *U*(−*d*,*d*), *d*=4,8,16,32. For the null pattern, we assign *μ*^0^ to *μ*^1^. For the scattered differential expression patterns, we are not concerned with the regulatory relationships. We choose entries of *μ*^1^ at random and add a random value sampled from *U*(−*d*,*d*) to them, as described in Algorithm 1. We remark that differential expression for *d*=4,8,16,32 corresponds to mean absolute log-differential expression $(\overline {|\Delta E|}) 2, 4, 8, 16$ across simulations, respectively. An average difference $\overline {|\Delta E|} = 16$ is extreme and unlikely to be seen in practice; as such it represents an upper limit to the context of gene expression.


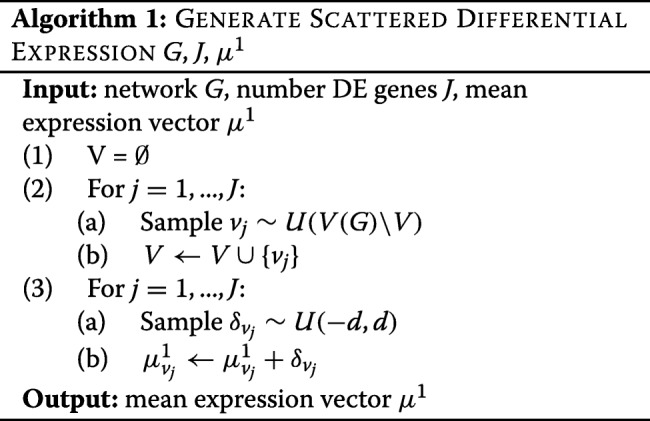


The generation of simulated coherent expression is more involved. We must first generate a subnetwork, and then generate gene expression in a way that ensures that differential gene expression is coherent in the subnetwork. To obtain a connected subnetwork for each module, we select a gene at random, and then select genes from the neighbourhood, growing the network iteratively as described in Algorithm 2.


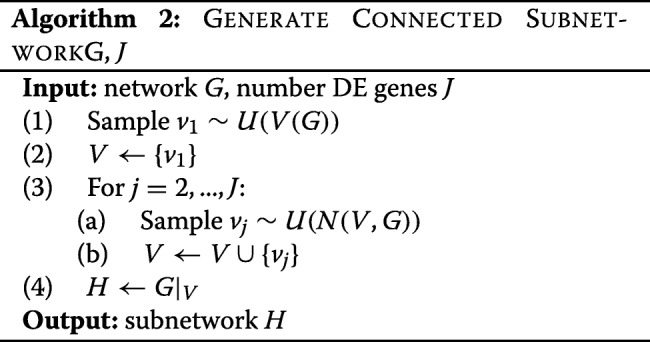


Here *N*(*V*,*G*) is the neighbourhood of the gene set *V* in network *G* and *G*|_*V*_ is the induced subnetwork of *G* with vertex set *V*. In the case of multiple modules, each module is created to be approximately the same size. Next, we generate mean log-expression vectors. This is done iteratively through gene set *V*(*H*), so that differential expression for gene *v*_*j*_ is coherent with at least one of its neighbours *v*_*k*_, *k*<*j*. This is described in Algorithm 3.


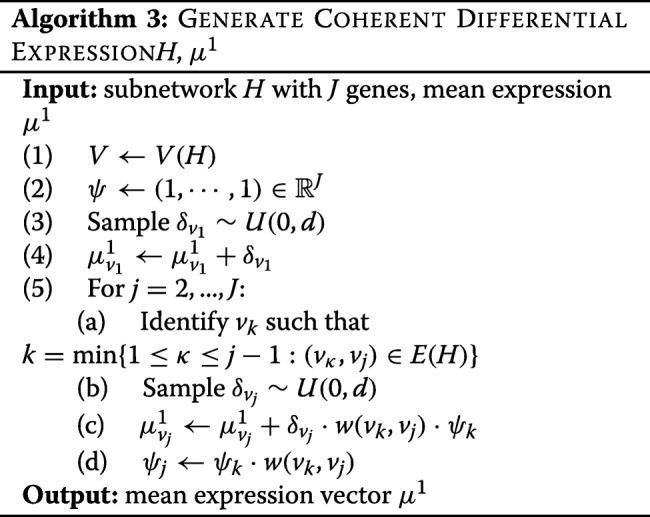


The vector of signs *ψ* contains the information on signs of differential expression for each gene. This is initialized as a vector of 1’s, and the sign for vertex *j* is adjusted as necessary so that its differential expression is coherent with at least one of the vertices *k*<*j*. Explicitly, the product *ψ*_*k*_·*w*(*v*_*k*_,*v*_*j*_) ensures that the change in expression for gene *v*_*j*_ is coherent with the change in expression for gene *v*_*k*_. At a minimum, there will be a simply connected subnetwork (i.e. a tree) connecting all DE genes. However, it is possible that two disconnected genes within this tree share a link within the larger network. If the link is promotional and the genes are both up- or both down-regulated, then this is coherent and the link is included in the coherent subnetwork. Similarly, if the link is inhibitory and one gene is up- while the other is down-regulated, then this is also coherent and the link is included in the coherent subnetwork.

#### Covariance structure

In our simulations, the covariance structure is informed by the graph structure of the network, as well as the nature of the link. In Eukaryotes, inducing links account for approximately 75 to 80% of regulators [20, 21]. McDonald et al. [20] report that the average proportion of activations for circadian networks is 0.74 in *Arabidopsis* and *Drosophila*, while generally for Eukaryotic signalling networks the average is 0.83. For each graph, we choose a random proportion of inducing links uniformly over (0.72,0.85), *p*∼*U*(0.72,0.85). We assign each link a relationship *w*_*jk*_ of 1 (non-inhibitory) with probability *p* and -1 (inhibitory) otherwise.

We construct a covariance matrix satisfying the conditional dependence structure of the network by first constructing the precision matrix **P**=**[***p*_*jk*_**]** from the adjacency matrix **A**=**[***a*_*jk*_**]** and then inverting to obtain the covariance matrix *Σ*=**P**^−1^, described in Algorithm 4.


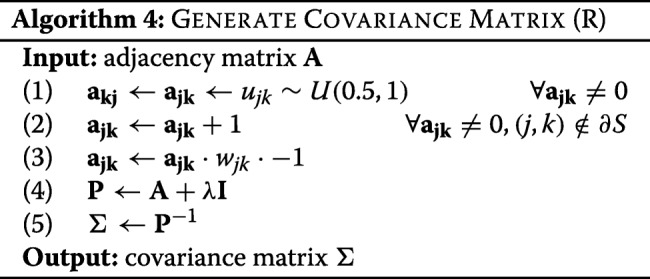


In step (1), we assign random values to link weights independently and identically distributed according to a uniform distribution *U*(0.5,1). In step (2), we add 1 to all non-boundary link weights, (*j*,*k*)∉*∂S*, ensuring that the genes in modules are more strongly coupled to each other than to the rest of the network. In step (3), we adjust all link weights by their relationship encoded in *w*_*jk*_, and multiply this by -1 to account for the relationship between partial correlation *ρ*_*jk*_ and the entries of the precision matrix *p*_*jk*_: $\rho _{jk}=-\mathbf {p_{jk}}/\sqrt {\mathbf {p_{jj}p_{kk}}}$. In step (4) we add a positive shift *λ***I** to ensure positive definiteness and hence ensure that we ultimately obtain a covariance matrix. The scale parameter *λ*=*λ*_1_+*λ*_2_ is calculated as follows. −*λ*_1_ is the smallest eigenvalue of the matrix **A** from step (3). It ensures positive semi-definiteness. We then calculate *λ*_2_>0 so that the resulting matrix has condition number equal to the number of genes *v* in the network. This ensures invertibility of the matrix. At this point, the matrix is a proper precision matrix. Finally in step (5) we obtain the covariance matrix. Thus by construction, this matrix is consistent with the network *G*, as described under “Gaussian graphical models” section.

#### Alternative methods

We compare CoNE to two alternatives, a standard network independent method and a network-constrained method. Both of these are implemented in R, a requirement placed in our search for methods.

LIMMA is a linear model based method that uses moderated t-statistics to assess the significance of the design as a predictor of gene expression [14]. LIMMA is a network-naive method. We include it as a baseline method in order to ascertain the improved inference resulting from incorporating network information. In our simulation study, we use LIMMA as the standard network-free method. We ascertain the significance of differences between log gene expression *δ*_*j*_ with Benjamini-Hochberg FDR (*α*=0.05). We keep the subnetworks corresponding to the genes identified as differentially expressed.

BioNet incorporates a network in the analysis of gene expression profiles for the detection of functional modules [22]. Thus BioNet represents a method with similar goals to CoNE. Beginning with a set of *p*-values assigned to each gene, a beta-uniform mixture model is fit, with the 1-parameter beta distribution *B*(*α*,1), *α*>0. Scores for each subnetwork are computed based on this model and an integer linear programming algorithm is used to locate the maximum scoring subnetwork. For our simulation study, we take the unadjusted *p*-values obtained for LIMMA and feed them into the BioNet algorithm with Benjamini-Hochberg FDR control (*α*=0.05). BioNet returns a connected subnetwork.

#### Evaluation of CoNE and alternatives on simulated data

Performance in simulations is evaluated via sensitivity (*SE*), specificity (*SP*), and precision (*P*) of the procedures to both genes and links. These are standard metrics, which in our notation are given as follows. Let *G* be the simulated regulatory network, *S* be the simulated coherent subnetwork, and $\widehat S$ the estimated coherent subnetwork. Then

$$\begin{aligned} {{SE}}_{genes}&\,=\,\frac{|V(\widehat S)\cap V(S)| }{|V(S)|}, & {{SE}}_{links}&\,=\,\frac{|E(\widehat S)\cap E(S)| }{|E(S)|}, \\ {{SP}}_{genes}&\,=\,\!1\!\,-\,\!\frac{|V(\widehat S)\backslash V(S)| }{|V(G)\backslash V(S)|}, & {{SP}}_{links}&\!\,=\,1-\frac{|E(\widehat S)\backslash E(S)| }{|E(G)\backslash E(S)|}, \\ {PR}_{genes}&=\frac{|V(\widehat S)\cap V(S)| }{|V(\widehat S)|}, & {PR}_{links}&=\frac{|E(\widehat S)\cap E(S)| }{|E(\widehat S)|}, \end{aligned} $$

where |·| counts the number of elements in a set.

In order to compactly evaluate the differences between CoNE and alternatives with respect to *SE* and *SP*, we fit generalized linear models to study the interactions between simulation parameters and inference procedures. Since the number of DE and non-DE genes is constant, *SE* and *SP* can be modelled according to a binomial distribution. *SE* and *SP* are thus modelled logistically as the interaction between inference method (*M*) and network topology (*T*; 0 for scale-free, 1 for exponential), network size (*N*; 0 for 500 genes, 1 for 2000 genes), differential expression pattern (*P*), mean absolute log-differential expression $(\overline {|\Delta E|})$, and sample size (*n*), where the linear predictor *η* is given by

7 $$\begin{array}{*{20}l} {}\eta=& \kappa \,+\, \mu M \,+\, \tau_{1}T \,+\, \nu_{1}N \,+\, \rho_{1}P\,+\, \lambda_{1}\log(\overline{|\Delta E|}) \,+\, \sigma_{1}\log(n)  \\ &\,+\,M\!\times\!(\tau_{2}T + \nu_{2}N + \rho_{2}P + \lambda_{2}\log(\overline{|\Delta E|}) + \sigma_{2}\log(n)).  \end{array} $$

The differential expression and sample size covariates were log-transformed to create a uniform spacing between consecutive parameters. This ensures that the high differential expression and high sample size cases do not have disproportionate leverage in the model. Since *PR* depends on the number of genes/links sampled, *PR* was modelled according to a negative binomial distribution, constructing the linear predictor as for *SE* and *SP*.

### Application

We consider an example gene expression experiment investigating particle-induced inflammation in pulmonary artery endothelial cells reported in Karoly et al. [23], specifically the effect of exposure to airborne ultrafine particles (UFPs) – particles with diameter less than 100 nm. The authors hypothesize that UFPs contribute to endothelial cell dysfunction by inducing transcriptional activation of genes involved in coagulation and inflammatory responses. To test this, they perform a cell culture study with one treatment group (exposure to 100 *μ*g/mL UFPs; n=4) and one control group (no UFP exposure; n=4) and measure the effects via gene expression.

#### Gene expression

Affymetrix microarray CEL files are downloaded from the Gene Expression Omnibus database [24], accession number GSE4567 [25]. Gene expression is corrected and normalized via the R-package oligo [26] using the default method and then log-transformed. Expression data is annotated with gene symbols using the R-package hgu133plus2.db [27]. Where gene symbols correspond to multiple expression values, we take the mean of the values within each sample.

#### Gene regulatory network

The human TRRUST V2 network [28], which is currently the most comprehensive public database for human regulatory interactions, is used as the seed network. This network consists of 800 transcription factors (TFs) and 2095 non-TFs, with 8444 regulatory links. We remove loops and multiple links; since this is a directed network, we treat links (*j*,*k*) and (*k*,*j*) as distinct. After restricting the gene expression dataset and TRRUST network to their common gene set, we obtain a GRN of 2731 nodes and 7966 links.

#### Differential expression

We infer the coherent subnetwork via CoNE with Benjamini-Hochberg control (FDR *α*=0.05). We also perform an updated analysis of the gene expression data with LIMMA, following Karoly et al. [23] to identify significant differentially expressed genes. We use updated annotation sources in order to ensure that differences between their procedures and CoNE reflect the methods and not the annotation source. Additionally, we perform a third analysis, following Karoly et al. [23] but this time restricting the gene set to those common to the GRN. In this way we can evaluate the marginal effect of using our procedure using a common set of genes. This provides some indication of how our method will perform when the full GRN becomes available.

#### KEGG pathway analysis

We perform gene set enrichment analyses of gene lists obtained for both the LIMMA and forward procedures, using the Human KEGG pathway database [29]. Gene sets are determined to be significantly enriched following Fisher’s exact test with Benjamini-Hochberg control (*α*=0.05).

## Results

### Simulations

**Fig. 4:**
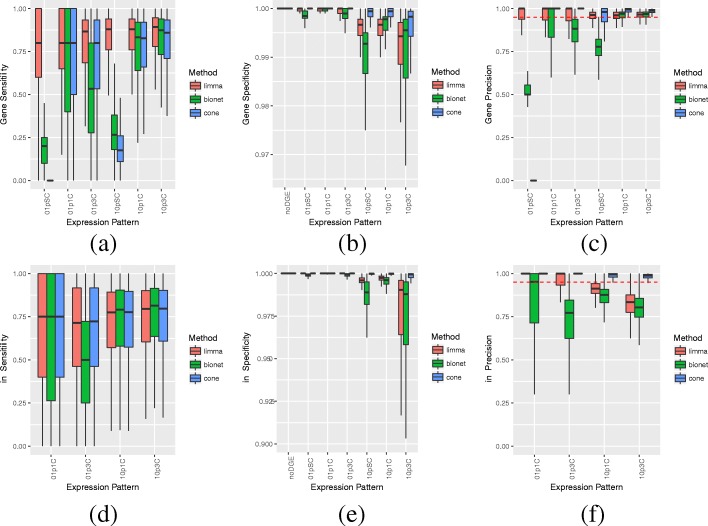
Performance of Differential Expression (DE) Procedures. We report the simulation results (100 replicates) for the three procedures with respect to (**a**) gene sensitivity, (**b**) gene specificity, (**c**) gene precision, (**d**) link sensitivity, (**e**) link specificity, and (**f**) link precision. Displayed are the results for 0% DE (noDGE), 1% scattered DE (01pSC), 1% DE in 1 module (01p1C), 1% DE in 3 modules (01p3C), 10% scattered DE (10pSC), 10% DE in 1 module (10p1C), 10% DE in 3 modules (10p3C). The dashed lines in Figures (**c**) and (**f**) indicate the 95% precision threshold. Outliers are omitted

CoNE is more specific and precise than LIMMA and BioNet (see Fig. 4b, c, e, f), at the expense of being less sensitive than LIMMA at the gene level and sometimes less sensitive than BioNet (Fig. 4a, d). CoNE is however more sensitive than LIMMA with respect to links.

CoNE controls the false discovery rate. In fact, CoNE controls error for genes better than either LIMMA or BioNet when differential gene expression presents as modules (Fig. 4c). CoNE does not control error for the 1%, scattered differential expression pattern because it does not detect genes in that scenario; this is by design. On the other hand, BioNet controls error poorly except for the case when a high proportion of genes are differentially expressed in modules. Further, CoNE controls error for links, even though it only explicitly controls gene error (Fig. 4f). Neither LIMMA nor BioNet control link error. Precision with respect to network topology and network size is reported in Table 1C. Method precision is not greatly affected by network topology or network size for any of the methods; this is as expected, since we are controlling for the false discovery rate.

**Table 1:** **Method Performance Estimates**

**A. Sensitivity**	Genes			Links		
	LIMMA	BioNet	CoNE	LIMMA	BioNet	CoNE
Topology	-0.002	-0.303	0.302	0.011	-0.360	-0.009
Network Size	-0.129	-0.608	-0.177	-0.188	-0.386	-0.186
**B. Specificity**	Genes			Links		
	LIMMA	BioNet	CoNE	LIMMA	BioNet	CoNE
Topology	-0.079	-0.151	-0.659	0.217	0.687	-0.0306
Network Size	0.497	1.025	0.865	1.266	1.253	0.970
**C. Precision**	Genes			Links		
	LIMMA	BioNet	CoNE	LIMMA	BioNet	CoNE
Topology	-0.000	-0.041	0.015	-0.020	-0.041	-0.003
Network Size	0.005	0.016	0.058	0.057	0.038	0.028

Network Topology (0 for scale-free, 1 for exponential) and network size (0 for 500 genes, 1 for 2000 genes) parameter estimates for the simulations from modeling method sensitivity and specificity by logistic regression and method precision by negative binomial regression

Sensitivity with respect to network topology and network size is reported in Table 1A. CoNE is more sensitive for genes for exponential networks versus scale-free networks, whereas the reverse holds for BioNet; LIMMA is indifferent. BioNet is less sensitive for links for exponential networks whereas CoNE and LIMMA are indifferent. All three methods are less sensitive with respect to genes and links as network size increases; note however that BioNet decreases more rapidly in sensitivity than either CoNE or LIMMA.

Specificity with respect to network topology and network size is reported in Table 1B. Specificity for both genes and links increases as network size increases for all three methods. Gene specificity is decreased for exponential networks relative scale-free networks. On the other hand, link specificity is increased for exponential networks relative scale-free networks for LIMMA and BioNet; CoNE is indifferent in this case.

The performance of the standard LIMMA procedure is nearly independent of the differential expression pattern, with strict error control over all patterns and topologies. The performance of CoNE and BioNet are more nuanced.

**Fig. 5:**
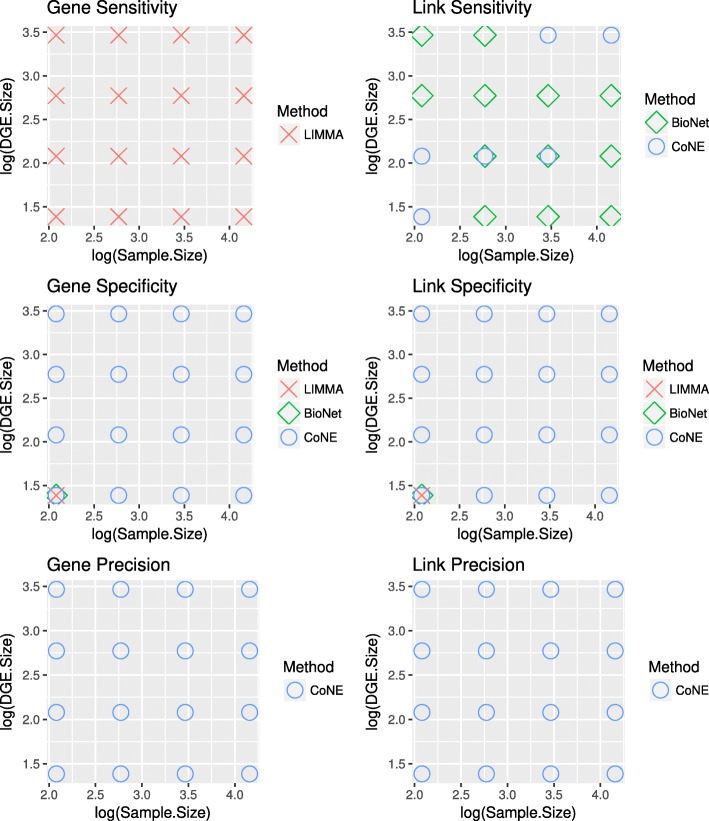
Median Method Performance. The performance by measure across log-sample size (*n*) and mean log-differential expression size (*ΔE*). The best method by median is presented for each (*n*,*ΔE*) pair

We investigated a range of sample sizes (*n*=8,16,32,64) and mean log-differential expression magnitudes (*ΔE*=2,4,8,16). The methods with the greatest performance for modular gene expression patterns in terms of median sensitivity, specificity, and precision are displayed in Fig. 5. It is clear that if gene sensitivity is the only measure of importance, LIMMA is the top performer across sample sizes *n* and differential expression magnitudes *ΔE*. On the other hand, if specificity or link sensitivity are important, then CoNE or CoNE and BioNet are better choices, respectively.

### Application: particle-induced inflammation

For the CoNE analysis of our example gene expression dataset, we obtained a coherent subnetwork with 80 genes and 119 links, with one large connected component with 76 genes, and 2 components consisting of gene pairs. Of the identified genes, 92.5% were up-regulated, indicating that the dominant response from exposure to ultrafine particles is activation. Of the identified links, 94% were inducing, whereas in the full GRN 77% were inducing. 51 KEGG pathways were identified as significantly enriched, of which 21 corresponded to non-infection, non-cancer, immune-related pathways. These are presented in Table 2.

**Table 2:** **Ultrafine particle-exposure enriched pathways**

Inflammation-related pathways	CO %	LI %
Antifolate resistance	14	—
Chemokine signaling	10	—
Cellular senescence	10	—
Fluid shear stress and atherosclerosis	15	—
Non-alcoholic fatty liver disease	10	—
Th17 cell differentiation	10	—
AGE-RAGE signaling (in diabetes)	15	18
IL-17 signaling	25	29
NF-kappa B signaling	14	19
NOD-like receptor signaling	15	22
Oxytocin signaling	17	20
Rheumatoid arthritis	14	19
TNF signaling	24	31
Cytokine-cytokine receptor interaction	—	14
Other immune-related pathways	CO %	LI %
B cell receptor signaling	15	—
Prolactin signaling pathway	9	—
T cell receptor signaling pathway	10	—
Th1 and Th2 cell differentiation	8	—
Cytosolic DNA-sensing	14	23
C-type lectin receptor signaling	17	25
Toll-like receptor signaling pathway	11	16
Leukocyte transendothelial migration	—	19
Oxidative stress-related pathways	CO %	LI %
FoxO signaling	12	—
HIF-1 signaling	—	16

The non-infection, non-cancer, immune-related KEGG pathways identified as enriched using Fisher’s exact test with Benjamini-Hochberg control (*α*=0.05). Columns CO % and LI % denote the proportion of genes identified for each pathway by CoNE and LIMMA approaches, respectively

The updated LIMMA-based analysis of [23] again yielded the Cytokone-cytokine receptor interaction, Wnt signaling, and MAPK signaling pathways as before, together with a number of other immune-related pathways. The analysis with the gene set restricted to those in common with the GRN returns the Cytokone-cytokine receptor interaction but not the other two pathways. This potentially reflects the imperfect matching between pathway databases and network knowledge. The list of significantly enriched pathways for the restricted analysis is presented in Table 2.

## Discussion

### Simulation

CoNE effectively identifies modules. CoNE has almost zero false positives in the null differential expression scenario. This suggests that if two distinct modules were truly differentially expressed, CoNE could not only identify them, but also separate them into two distinct connected components. Indeed, results for scattered and modular differential expression confirm this. Whereas CoNE does not typically obtain large connected components for scattered differential expression, but only a few small components of two or three genes, CoNE correctly identifies modules in the modular differential expression scenario. Thus differentially expressed modules identified by CoNE constitute actual findings.

BioNet’s variation in sensitivity, specificity, and precision is large in comparison to CoNE and LIMMA. Additionally, BioNet has some difficulties with large sample sizes or large absolute mean differential expression, sometimes failing to identify any signals. There is perhaps difficulty in fitting the beta-mixture distribution using maximum-likelihood estimates when the signal is very strong. When some *p*-values are numerically close to 1, the log-likelihood for the beta distribution *B*(*α*,1) is not well-defined, while when some *p*-values are numerically close to 0, the log-likelihood for the beta distribution *B*(1,*β*)(*β*>0) is not well-defined [30].

Across all our simulations, we find that CoNE is less sensitive to differential gene expression than LIMMA. However, for these simulations we do not apply a ‘relevance’ threshold, such as at least 2-fold change, to the differential expression submitted for LIMMA analysis. It is typical to use a threshold on the fold change to classify differential expression as relevant. Thus truly differentially expressed genes whose mean differential expression falls below the threshold are necessarily excluded, reducing the sensitivity of LIMMA in practice. It may be that CoNE as applied is more sensitive than LIMMA as it is typically applied. In a sense, we have replaced the relevance threshold with coherence status in defining relevance.

We chose to simulate differential expression via GGMs in order to avoid conflating performance with emergent behaviour in a system. We had to address the dual questions, “What level of complexity is sufficient” and “What level of complexity is too much?” Using GGMs allows us direct control over generating coherent differential expression, so that we can determine whether the method is able to use coherent expression to detect functional modules to greater precision over alternatives. We are able to show that if there is coherent differential expression, then indeed, our method is able to detect functional modules. We judge this sufficient. Had we simulated our data in a more complex manner, for example using Markov chains, it is not clear how to separate the differentially expressed functional modules from the surrounding network without severing all the connections. This would destroy any covariation between the functional module and the rest of the network. Further, it is not clear how to simulate ‘null’ differential expression in the non-differentially expressed subnetwork. Now that we have shown that, yes, CoNE detects differentially expressed functional modules, we can apply it to simulations of dynamic systems, such as those generated via Markov chains, to detect functional modules that arise as emergent properties of the structure of the regulatory network. That would be very interesting, but outside the scope of the current article. Essentially, we have used GGMs to avoid conflating performance with emergent behaviour in a system.

### Particle-induced inflammation

CoNE identified 13 inflammation-related pathways, 1 oxidative stress-related pathway, and 7 other immune-related pathways, while the restricted network-agnostic procedure (LIMMA) identified 8 inflammation-related pathways, 1 oxidative stress-related pathway, and 4 other immune-related pathways. The identification of oxidative stress pathways is consistent with the hypothesis that air-borne particles induce inflammatory response through an oxidative stress mechanism. Karoly et al. [23] hypothesized an increase in inflammatory responses. That use of CoNE identifies two-fold more inflammation-related pathways, overlapping with most pathways identified for the LIMMA-based analysis, indicates an increase in sensitivity at the level of gene set enrichment. Using CoNE allows us to identify these pathways because it is more specific at the level of gene expression.

**Fig. 6:**
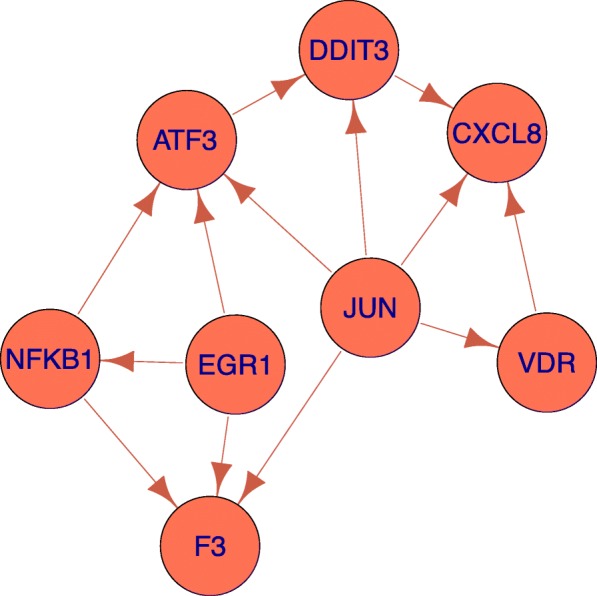
Subnetwork of coherent network in ultrafine particle-exposure study (FDR *α*=0.01). The transcription regulatory subnetwork of human pulmonary endothelial cells undergoing coherent differential expression from exposure to ultrafine particles, restricted to eight genes. All genes increased in expression

**Fig. 7:**
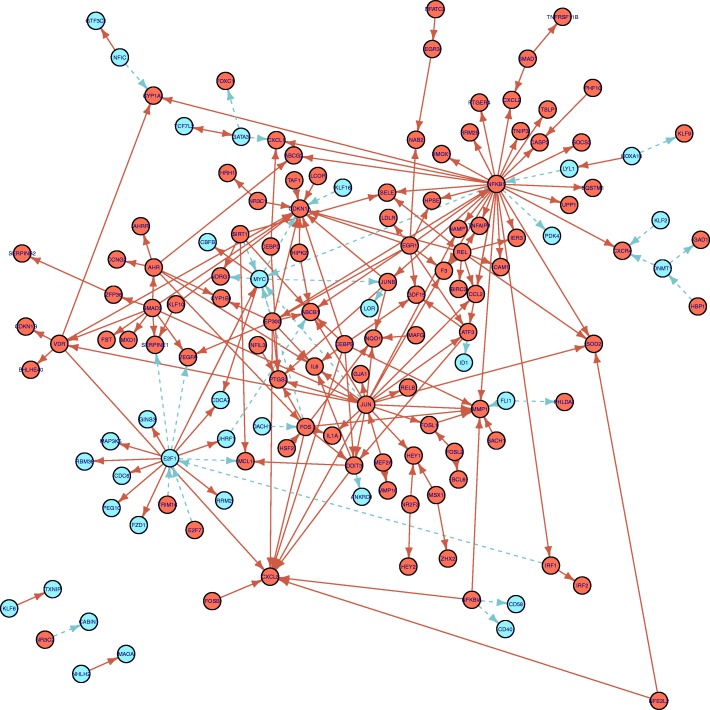
Coherent network in ultrafine particle-exposure study (FDR *α*=0.05). The transcription regulatory subnetwork of human endothelial cells undergoing differential expression from exposure to ultrafine particles. Gene expression both increased (circles) and decreased (squares). Inducing and inhibiting links are represented as solid and dashed arrows, respectively

Karoly et al. [23] are particularly interested in tissue factor (TF), noting that its gene expression (*F*3) is up-regulated. In an additional experiment, they find that increased TF protein induces increased release of the cytokine IL-8 (gene *CXL*8). While the coherent subnetwork does not reveal a directed path *F*3 to *CXL*8, we observe that *EGR*1, *JUN*, and *NFKB*1 appear to be inducing *F*3. Genes *EGR*1, *JUN*, and *NFKB*1 have directed paths leading to *CXL*8. The structure of this network suggests a synergy between *EGR*1, *JUN*, and *NFKB*1 resulting in the increased expression of *CXL*8. This subnetwork is presented in Fig. 6. A high resolution figure of the full coherent subnetwork is presented in Fig. 7.

## Conclusion

CoNE infers differentially activated modules in gene regulatory networks. While this work is developed in the context of gene regulatory networks, the methodology is sufficiently general for application to other molecular networks.

None of the three methods CoNE, BioNet, or LIMMA uniformly dominates any other across all measures. When it is important that error be well-controlled, the CoNE and LIMMA procedures are most appropriate. There are some scenarios where BioNet outperforms the rest; however, BioNet’s extreme variation in sensitivity is hard to predict. Further, it begins to perform poorly when the signal to noise ratio is high, or when the sample size is high. We recommend comparing network-constrained approaches to a LIMMA baseline. The disparity between these can indicate whether we are observing a scattered or modular differential expression regime. Further, it can safeguard against high variation in precision or sensitivity. Finally, current network knowledge is incomplete, so that using only network-dependent methods will miss potentially important signals.

Different experimental settings are represented by combinations of sample size and differential expression magnitude. In vitro cell line studies generally fall under the low sample size, high magnitude setting, represented in the upper left corners of the plots in Fig. 5. Since in vitro experiments can be well-controlled, the signal-to-noise ratio is high, which is here represented by high differential expression magnitude. On the other side, successful in vivo studies in humans generally have many biological replicates, which is necessary due to the uncontrollable nature of study participants, resulting in lower signal-to-noise ratio, represented in the lower right corners of the plots in Fig. 5. The simulations have not provided an unambiguous top performer. When detecting novel signals, we must trade between gene and link sensitivity, and hence between LIMMA and CoNE. However, when our goal is mechanistic understanding, a method with great specificity is needed, and hence CoNE is recommended.

Network-constrained approaches to identifying regulated features are accompanied with the desire for increased power and precision to detect the truly relevant changes before an expansive, noisy background. We observe in these simulations that CoNE is highly specific, and has greater power for detecting links, while LIMMA retains greatest power for detecting genes. However, the network-constraints at the same time bias our possible results. It may be that the non-coherent differential signals in the data point to interesting regulatory changes. These could represent cases where the actual regulatory structure has changed. Such an interpretation would rely on the validity of the underlying network model, and so it would be worthwhile to investigate how errors in the underlying network could affect the analysis of functional modules. This is however outside the scope of this article. Thus, it is worthwhile to investigate both coherent and non-coherent differential regulatory signals.

## Abbreviations

CDE: Coherent differential expression
CoNE: Coherent network expression
DE: Differentially expressed
FDR: False discovery rate
GGM: Gaussian graphical model
GRN: Gene regulatory network
PR: Precision
SE: Sensitivity
SP: Specificity
UFP: Ultrafine particle

## References

[1] Barabási A-L, Oltvai ZN (2004). Network biology: understanding the cell’s functional organization. Nat Rev Genet.

[2] Ravasz E, Somera AL, Mongru DA, Oltvai ZN, Barabási AL (2002). Hierarchical organization of modularity in metabolic networks. Science.

[3] Friedman J, Hastie T, Tibshirani R (2008). Sparse inverse covariance estimation with the graphical lasso. Biostatistics.

[4] Margolin AA, Nemenman I, Basso K, Wiggins C, Stolovitzky G, Favera RD, Califano A. ARACNE: An Algorithm for the Reconstruction of Gene Regulatory Networks in a Mammalian Cellular Context. BMC Bioinf. 2016;7(1). 10.1186/1471-2105-7-S1-S7. 10.1186/1471-2105-7-S1-S7 PMC1810318 16723010

[5] Warsow G, Greber B, Falk SS, Harder C, Siatkowski M, Schordan S, Som A, Endlich N, Schöler H, Repsilber D, Endlich K, Fuellen G. ExprEssence - Revealing the essence of differential experimental data in the context of an interaction/regulation net-work. BMC Syst Biol. 2010;4(164). 10.1186/1752-0509-4-164 PMC3012047 21118483

[6] Woo JH, Shimoni Y, Yang WS, Subramaniam P, Iyer A, Nicoletti P, Rodríguez Martínez M, López G, Mattioli M, Realubit R, Karan C, Stockwell BR, Bansal M, Califano A (2015). Elucidating Compound Mechanism of Action by Network Perturbation Analysis. Cell.

[7] Ernst M, Du Y, Warsow G, Hamed M, Endlich N, Endlich K, Escobar HM, Sklarz L-M, Sender S, Junghanß C, Möller S, Fuellen G, Struckmann S (2017). FocusHeuristics - expression-data-driven network optimization and disease gene prediction. Sci Rep.

[8] Hill SM, Nesser NK, Johnson-Camacho K, Jeffress M, Johnson A, Boniface C, Spencer SEF, Lu Y, Heiser LM, Lawrence Y, Pande NT, Korkola JE, Gray JW, Mills GB, Mukherjee S, Spellman PT (2017). Context Specificity in Causal Signaling Networks Revealed by Phosphoprotein Profiling. Cell Syst.

[9] Li C, Li H (2008). Network-constrained regularization and variable selection for analysis of genomic data. Bioinformatics.

[10] Sun H, Wang S (2012). Penalized logistic regression for high-dimensional DNA methylation data with case-control studies. Bioinformatics.

[11] Avey S, Mohanty S, Wilson J, Zapata H, Joshi SR, Siconolfi B, Tsang S, Shaw AC, Kleinstein SH (2017). Multiple network-constrained regressions expand insights into influenza vaccination responses. Bioinformatics.

[12] Ma J, Shojaie A, Michailidis G (2016). Network-based pathway enrichment analysis with incomplete network information. Bioinformatics.

[13] Lenth RV (2016). Least-squares means: The R package lsmeans. J Stat Softw.

[14] Ritchie ME, Phipson B, Wu D, Hu Y, Law CW, Shi W, Smyth GK (2015). limma powers differential expression analyses for RNA-sequencing and microarray studies. Nucleic Acids Res.

[15] Benjamini Y, Hochberg Y (1995). Controlling the False Discovery Rate: A Practical and Powerful Approach to Multiple Testing. J Roy Stat Soc Ser B.

[16] Rush S, Repsilber D. Capturing context-specific regulation in molecular interaction networks. bioRxiv:10.1101/254730. 2018. https://www.biorxiv.org/content/early/2018/01/29/254730.full.pdf. 10.1101/254730. https://www.biorxiv.org/content/early/2018/01/29/254730. 10.1186/s12859-018-2513-7 PMC6303932 30577761

[17] Kolaczyk ED, Csárdi G (2014). Statistical Analysis of Network Data with R.

[18] Albert R, Barabási A-L (2002). Statistical mechanics of complex networks. Rev Mod Phys.

[19] Csardi G, Nepusz T (2006). The igraph software package for complex network research. InterJournal.

[20] McDonald D, Waterbury L, Knight R, Betterton MD. Activating and inhibiting connections in biological network dynamics. Biol Direct. 2008;3(49). 10.1186/1745-6150-3-49. 10.1186/1745-6150-3-49 PMC2651858 19055800

[21] Wang T, Feng Y, Wang Q. PAIRS: Prediction of Activation/Inhibition Regulation Signaling Pathway. Computational Intell Neurosci. 2017. 10.1155/2017/7024516. 10.1155/2017/7024516 PMC5392402 28469669

[22] Beisser D, Klau GW, Dandekar T, Müller T, Dittrich MT (2010). BioNet: an R-Package for the functional analysis of biological networks. Bioinformatics.

[23] Karoly ED, Li Z, Dailey LA, Hyseni X, Huang Y-CT (2007). Up-regulation of Tissue Factor in Human Pulmonary Artery Endothelial Cells after Ultrafine Particle Exposure. Environ Health Perspect.

[24] Barrett T, Wilhite SE, Ledoux P, Evangelista C, Kim IF, Tomashevsky M, Marshall KA, Phillippy KH, Sherman PM, Holko M, Yefanov A, Lee H, Zhang N, Robertson CL, Serova N, Davis S, Soboleva A (2013). NCBI GEO: archive for functional genomics data sets - update. Nucleic Acids Res.

[25] Karoly E, Huang Y. Endothelial cell culture with Chapel Hill Ultrafine particle. GEO accession GSE4567; Karoly et al. 2007. https://www.ncbi.nlm.nih.gov/geo/query/acc.cgi?acc=GSE4567. Accessed 2018-04-09.

[26] Carvalho BS, Irizarry RA (2010). A framework for oligonucleotide microarray preprocessing. Bioinformatics.

[27] Carlson M. Hgu133plus2.db: Affymetrix Human Genome U133 Plus 2.0 Array Annotation Data (chip Hgu133plus2). 2016. R package version 3.2.3. https://bioconductor.org/packages/release/data/annotation/html/hgu133plus2.db.html.

[28] Han H, Cho J-W, Lee S, Yun A, Kim H, Bae D, Yang S, Kim CY, Lee M, Kim E, Lee S, Kang B, Jeong D, Kim Y, Jeon H-N, Jung H, Nam S, Chung M, Kim J-H, Lee I (2018). TRRUST v2: an expanded reference database of human and mouse transcriptional regulatory interactions. Nucleic Acids Res.

[29] Kanehisa M, Sato Y, Kawashima M, Furumichi M, Tanabe M (2016). KEGG as a reference resource for gene and protein annotation. Nucleic Acids Res.

[30] Schröder C, Rahmann S. A hybrid parameter estimation algorithm for beta mixtures and applications to methylation state classification. Algoritm Mol Biol. 2017;12. 10.1186/s13015-017-0112-1. 10.1186/s13015-017-0112-1 PMC5563068 28828033

